# Current Issues in Fungal Infections and COVID-19

**DOI:** 10.3390/jof8111115

**Published:** 2022-10-23

**Authors:** Ana Fernández-Cruz, Eleni Magira

**Affiliations:** 1 Infectious Diseases Unit, Internal Medicine Department, University Hospital Puerta de Hierro-Majadahonda, Health Research Institute Puerta de Hierro-Segovia de Arana, 28222 Majadahonda, Spain; 2 First Department of Critical Care Medicine and Pulmonary Services, Evangelismos Hospital, National and Kapodistrian University of Athens Medical School, 11527 Athens, Greece

The COVID-19 pandemic has brought up a new host for fungal invasive infections. Patients with viral pneumonia caused by SARS-CoV-2 present an unexpectedly high rate of invasive fungal infections. This Special Issue of *Journal of Fungi* focuses on the latest research findings in this complex relationship, including pathogenesis, epidemiology and the outcome of post-SARS-CoV2 fungal infections. Moreover, it addresses data about the particular issues of COVID-19-associated invasive pulmonary aspergillosis (CAPA) including among others specific risk factors, differences and similarities compared to other viral–fungal interactions and the differentiation of colonization and infection.

Currently, a large number of publications on CAPA have come to light, but there is still room for clarification in many areas. Calderón-Parra J et al. [1] illustrate the association between CMV and CAPA. CMV is a well-known risk factor for aspergillosis in severely immunocompromised patients. According to the results of this case–control study, CMV replication was associated with CAPA and could potentially herald the diagnosis of CAPA. Peláez-García de la Rasilla et al. [2] examined in a prospective study with respect to whether the hospital environment was the source of the aspergillosis in CAPA patients. This is the first study monitoring and genotyping *A. fumigatus* isolates obtained from hospital air and from COVID-19 patients admitted with aspergillosis. Contrary to the intuitive assumption that CAPA is a nosocomial infection, their genotypic analysis of environmental and clinical samples suggests that the acquisition of *A. fumigatus* by COVID-19 patients may not occur in hospitals. The report from Sivasubramanian G et al. [3] of a large cohort of COVID-19 patients diagnosed of CAPA by means of 2020 ECMM/ISHAM consensus criteria for CAPA adds information on the epidemiology and outcomes of this disease.

The incidence of COVID-19-associated mucormycosis (CAM) has been dramatically high in areas such as India, particularly in the form of rhino-orbito-cerebral mucormycosis (ROCM). Bilgic A et al. [4] inform us about its risk factors and characteristics from an ophthalmologist perspective, and Janjua OS et al. [5] complement their information with a review from a maxillofacial standpoint. Treating fungal infections in patients with COVID-19 in a timely manner, whilst avoiding the unnecessary use of antifungals, is challenging. A delay in diagnosis is considerable even with ROCM involvements. Pulmonary mucormycosis might be underdiagnosed, and a delay in diagnosis is even more prevalent than in facial presentations due at least in part to the lack of rapid diagnostic techniques for Mucorales. Two studies in this Special Issue explore the usefulness of molecular techniques for the diagnosis of mucormycosis, especially in low-income areas. Mohapatra S et al. [6] evaluate a pan-fungal PCR based on primers targeting the 28S large subunit rRNA gene, both in nasal swabs and surgical biopsies of suspected ROCM cases, confirming an earlier identification of the fungus and an increased sensitivity when added to fungal stains even in the case of performing only endonasal swabs. A PCR assay identified mixed infections in 17 of 33 (52%) patients, of which 6 (35%) were KOH + CFW negative and 7 (41.2%) were culture negative. The diagnosis would have been missed in this large proportion of patients had it been based only on smear or culture reports. Next, Davies G et al. [7] report a potential novel opportunity for the non-invasive detection of mucormycosis caused by *Rhizopus arrhizus* (formerly *Rhizopus oryzae*), the principal agent of the disease worldwide. Specifically, they developed a lateral flow device for *Rhizopus arrhizus* based on a specific monoclonal antibody raised against an extracellular polysaccharide (EPS) antigen from *Rhizopus arrhizus* for applications in both human serum and human BALf. Their encouraging results now require validation in the clinic to determine its utility in human disease.

The management of both CAPA and CAM has been challenging due to the need for achieving adequate exposure to effective antifungals in COVID-19 patients who are often hospitalized in the ICU with concomitant renal replacement therapy or extracorporeal membrane oxygenation, and are frequently obese. Isavuconazole could provide some advantages over other first line antifungals. Ullah N et al. [8] provide a review of the literature and guide us through tricky scenarios where therapeutic drug monitoring would be helpful and underline the advantages of isavuconazole use in this setting.

COVID-19 not only has been associated with an increase in mold infection but it has also been associated with candidemia. The article by Machado M et al. [9] in the current Special Issue confirms that candidemia incidence increased in COVID-19 patients, but genotyping shows that this increase is not due to uncontrolled intrahospital transmission. However, the study by Ramos-Martínez A et al. [10] is a warning about the risk of a transmission of resistant microorganisms among COVID-19 patients as it highlights the impact of an emerging fungal pathogen: fluconazole-resistant *Candida parapsilosis*. Further dedicated studies are needed to inform appropriate preventive measures that need to be undertaken.

Described less often than CAPA, *Pneumocystis jirovecii* infection has been associated with COVID-19 as well. Gioia F et al. [11] present a systematic review of previously published cases and propose a distinction between different patterns of concurrent SARS-CoV-2 and *P. jirovecii* infection in regards to colonization, superinfection and coinfection.

Finally, two years into the COVID-19 pandemic, the narrative review by Casalini et al. [12] provides a comprehensive picture of the different invasive fungal infections that have been described to complicate COVID, from the widely studied CAPA to the less frequently reported post-COVID-19 cryptococosis or pneumocystosis. Due to diagnostic difficulties and differences in criteria, the true incidence of fungal infection in patients with COVID-19 is unclear. Interestingly, the authors perform a critical analysis of the difficulties of diagnosing CAPA and distinguishing it from colonization via the use of the diverse available algorithms.

In summary, we are confident that this Special Issue is a valuable collection of articles that will be of interest for all those seeking up-to-date information on COVID-19 associated fungal infections. We highly appreciate the efforts of all the authors and reviewers that made the publication of this Special Issue on fungal infections and COVID-19 possible.
